# Cellular mechanisms of cyclophosphamide-induced taste loss in mice

**DOI:** 10.1371/journal.pone.0185473

**Published:** 2017-09-26

**Authors:** Nabanita Mukherjee, Shreoshi Pal Choudhuri, Rona J. Delay, Eugene R. Delay

**Affiliations:** Department of Biology and Vermont Chemosensory Group, University of Vermont, Burlington, Vermont, United States of America; Duke University, UNITED STATES

## Abstract

Many commonly prescribed chemotherapy drugs such as cyclophosphamide (CYP) have adverse side effects including disruptions in taste which can result in loss of appetite, malnutrition, poorer recovery and reduced quality of life. Previous studies in mice found evidence that CYP has a two-phase disturbance in taste behavior: a disturbance immediately following drug administration and a second which emerges several days later. In this study, we examined the processes by which CYP disturbs the taste system by examining the effects of the drug on taste buds and cells responsible for taste cell renewal using immunohistochemical assays. Data reported here suggest CYP has direct cytotoxic effects on lingual epithelium immediately following administration, causing an early loss of taste sensory cells. Types II and III cells in fungiform taste buds appear to be more susceptible to this effect than circumvallate cells. In addition, CYP disrupts the population of rapidly dividing cells in the basal layer of taste epithelium responsible for taste cell renewal, manifesting a disturbance days later. The loss of these cells temporarily retards the system’s capacity to replace Type II and Type III taste sensory cells that survived the cytotoxic effects of CYP and died at the end of their natural lifespan. The timing of an immediate, direct loss of taste cells and a delayed, indirect loss without replacement of taste sensory cells are broadly congruent with previously published behavioral data reporting two periods of elevated detection thresholds for umami and sucrose stimuli. These findings suggest that chemotherapeutic disturbances in the peripheral mechanisms of the taste system may cause dietary challenges at a time when the cancer patient has significant need for well balanced, high energy nutritional intake.

## Introduction

Changes in taste functions are among the most common side effects suffered by cancer patients treated with chemotherapeutics. Recent studies report the prevalence of taste disturbances in chemotherapy patients is high, ranging from 65 to 80% [1–5]. These disturbances are usually in the form of hypogeusia (decreased sensitivity), dysgeusia (distortion of taste), or ageusia (absence of taste) [6–9]. These changes can be a major concern during cancer treatment because they can lead to lower food intake at a time when energy demands are high, thus resulting in malnutrition, slower recovery and poorer quality of life for the afflicted patients [4, 10–18]. However, little is known about how or why chemotherapy causes taste disturbances.

A long-standing explanation for the chemotherapy-associated changes in taste functions is that exposure to these drugs induces conditioned taste aversions (CTA). By this explanation, patients associate drug-induced nausea and vomiting with foods they consumed during and/or after chemotherapy administration, thus acquiring a conditioned taste aversion for those foods [19–22]. While this phenomenon may occur, our previous reports using a mouse model suggest that chemotherapy drugs have much more direct disruptive effects on the taste system. We evaluated the effects of a single injection of cyclophosphamide (CYP), a widely-used chemotherapy drug, on the functionality of the taste system. Behavioral experiments using umami or sucrose stimuli, revealed a two-phase disturbance in taste acuity and loss of taste sensitivity of mice after a single IP injection of CYP. The first disturbance occurred immediately after injection and lasted up to 5 days post-injection, and a second disturbance occurred between 8–15 days post-injection [23, 24]. In addition to the bi-phasic behavioral effects, histological assays of fungiform and circumvallate taste buds and Von Ebner glands [24] suggested CYP has direct effects on taste epithelium, including reducing the number of fungiform papillae by 40–50% within 4 days after injection. However, circumvallate taste buds did not decrease in number or show a loss of cells until 8–12 days after injection [23–25]. In addition, it appeared that cell proliferation in the basal layer of tongue epithelium might also be adversely affected by CYP. These initial findings suggest CYP has a distinctive biological impact on the taste system that might account for disturbances in taste with chemotherapy.

This study was intended to extend our understanding of the effects of CYP on the taste system. The sense of taste is primarily mediated by taste sensory cells (TSCs) located in the taste buds of the tongue. These taste buds are found in three different areas of the tongue: anterior (fungiform papillae), posterior (circumvallate papillae) and posterolateral (foliate papillae). Fungiform taste buds are on the surface of the tongue while circumvallate and foliate taste buds are in the walls of their respective crypts. Taste buds in mice are comprised of approximately 50–100 cells of four morphologically and functionally distinct cells types: TSC Types I-III and post-mitotic, precursor cells which are also called Type IV cells [26, 27]. Type II TSCs are believed to have an average lifespan of 8–14 days whereas Type III TSCs appear to have an average lifespan of 22 days [28, 29]. Type I TSCs may have two sub-populations of cells, one with a lifespan of about 8 days and the second over 22 days. All four cell types are renewed continuously [30, 31]. Cells regarded as progenitor cells are located in the basal layer just outside the taste bud [30, 32–36]. These basal cells divide asymmetrically to produce transit amplifying cells [29, 35] which give rise to the eventual mature, specialized TSCs [29, 30, 33, 34, 37]. Type I cells are glial-like cells that extend lamellate processes around other cells. They express ROMK, NTPDase 2 and the glutamate–aspartate transporter GLAST [38–40] and may play a role in salt taste transduction [41]. Type II cells have a characteristic large, round nucleus and are the main receptor cells responsible for detection of sweet, bitter and umami stimuli [42–45]. These cells express all the transduction elements associated with G-protein-coupled transduction linked to a phospholipase C-β2 (PLCβ2) second messenger signaling pathway [46, 47] and utilize ATP as a neurotransmitter [26]. Type III cells, sometimes called presynaptic cells [48], make synaptic contacts with the gustatory fibers and express synaptic proteins such as SNAP-25 [49], NCAM [50], Car4 [51], and serotonin [52]. They are thought to be responsible for detection of sour stimuli [53] and appear to be involved in salt detection [54]. Understanding how CYP affects these cells and the processes underlying their replacement could lead to methods for protecting these cells from chemotherapy drugs and other cytotoxic agents.

This study was designed to expand on our previous findings and explore further the effects of CYP on the taste system and the underlying mechanisms behind these effects using several immunohistochemical assays. We hypothesize that chemotherapy drugs such as CYP target proliferating cells of the taste bud and interrupt the timely renewal of TSCs. Our data suggest that CYP has a cytotoxic effect on the taste system, which kills normal taste cells as well as the progenitor cells in taste epithelium and leads to changes in the normal taste cell replacement cycle.

## Materials and methods

### Ethical consideration

This study followed strict accordance with recommendations in the Guide for the Care and Use of Laboratory Animals of the National Institutes of Health. All experimental procedures were reviewed and approved by the University of Vermont’s Institutional Animal Care and Use Committee (IACUC protocol: 14–003). Mice were euthanized by sodium pentobarbital followed by transcardial perfusion. All efforts were made to minimize suffering.

### Animals

Male C57BL/6J mice, obtained from Jackson Laboratory (Bar Harbor, ME), were housed in groups of 2–5 mice. They were at least 3 months old and weighed between 25–32 g. Food (Purina Mouse Chow, Prolab RMH 3000) and water were available *ad libitum*. The mouse colony was maintained on a regular 12/12 hr light-dark cycle.

### CYP and saline injections

CYP (Cyclophosphamide monohydrate, Acros Organics, New Jersey, USA) solutions were prepared fresh every day in saline. The 75 mg/kg body weight (IP) dose was chosen after a dose-response experiment with three different doses (37.5, 75 and 150 mg/kg body weight). At 150 mg/kg or greater, CYP can cause nephrotoxicity [55] whereas the effects of 37.5 mg/kg were more difficult to detect in the taste system. Therefore, 75mg/kg was selected for the study as the dose was not lethal, yet triggered a detectible response in the taste epithelium. It also allowed us to relate the current findings to our initial behavioral experiments [23–25]. The control group received a saline injection at the same volume/kg body weight.

### Sample collection

The day in which a subject was injected either with CYP or saline, was referred to as day 0. All mice were randomly assigned to be euthanized either on the day of injection or on day 4, 8, 10, 12 or 16 post-injection to collect the tongue for further processing. Mice assigned to the cell-death assays were similarly assigned to their time points for euthanasia measured in hours post-injection. Following euthanasia, transcardial perfusion was conducted using Heparin-1X in phosphate buffered saline (PBS) and 4% paraformaldehyde mixed in 0.1 M PBS. Tongues were dissected and fixed in the 4% paraformaldehyde solution overnight at 4^0^ C. After cutting the tongue into halves, the tissues were passed through a sucrose gradient (0.5, 1 and 1.5 M) for cryo-protection before being embedded in OCT compound (Tissue Tek, Sakura Finetec USA Inc., Torrence, CA) and stored at -80^o^ C until cryo-sectioned at a thickness of 20 μm. Tissue sections were mounted directly on slides and stored at -80^o^ C until processing.

### Cell death assays

#### Terminal deoxynucleotidyl transferase-mediated dUTP nick end labeling (TUNEL) assay

TUNEL and caspase-3 assays have shown that CYP can cause cell death in urinary bladder epithelium [56]. To test whether CYP exerts the same effects in taste epithelium, TUNEL assays were conducted on fungiform and circumvallate sections at 0, 2, 4, 6, 8, 12, 18 and 24 hrs post injection. TUNEL was done using *In Situ* Cell Death Detection kit TMR red (Cat# 12 156 792 910, Roche Applied Science, Indianapolis, IN, USA) following the manufacturer’s recommended protocol. Briefly, after washes in PBS and blocking with 3% H_2_O_2_ in methanol, slides were incubated with 0.1 M sodium citrate buffer (pH 6.0) at 91^0^ C for 15 min. The slides were cooled to room temperature and permeabilized in freshly prepared 0.1% sodium citrate mixed with 0.1% triton X-100 for 2 min on ice. After PBS wash, the sections were incubated in blocking solution (20% normal goat serum (NGS), 3% bovine serum albumin, in 50mM Tris-HCl, pH 7.5) for 30 min at room temperature. The sections were washed with PBS and incubated for 1 hr at 37^0^ C with the kit reaction mixture. Sections were washed again in PBS and double labeled with the nuclear marker Sytox green (1:30,000, Cat # S7020, Molecular Probes, Eugene, OR, USA) before mounting cover slips with Fluoromount G (#0100–001, Southern Biotech, Burmingham, AL, USA).

#### Cleaved caspase-3 assay

Since TUNEL can label fragmented DNA from either necrotic activity or apoptosis, we looked at cleaved caspase-3 as a marker that detects cells in a specific phase of apoptosis. The role of caspase-3 in apoptosis is to cleave and activate caspases-6, 7 and 9 prior to the apoptotic cell breaking down before removal [57]. Cleaved caspase-3 assays were conducted on tissues at 0, 2, 4, 6, 8, 12, 18, 24, 36 and 48 hrs post injection. The protocol was adapted from French et al. [58]. Briefly, the slides were exposed to 200ug/ml proteinase K in PBS for 15 min at 37^0^ C for antigen retrieval. The sections were exposed to rabbit anti-cleaved caspase-3 primary antibody (Cat# 9661S, Cell Signaling, Danvers, MA, USA; RRID: AB-2341188) at a concentration of 1:100 overnight at 4^0^ C. After washing in PBS, the sections were incubated in Alexa 546 goat-anti-rabbit secondary antibody (1:1000 dilution, Cat# A-11010, Invitrogen, ThermoFisher Scientific, Waltham, MA, USA; RRID: AB-2534093) for 2 hrs at room temperature. The tissues were then counter-reacted with Sytox green.

### Cell proliferation experiments

We focused on two different areas of the tongue, the anterior part which contains fungiform taste buds scattered on the surface of the tongue and the posterior part which has circumvallate papilla containing taste buds along the walls of its crypt. In addition, we examined the lingual epithelium to see if the proliferating cells of non-taste epithelium react differently to CYP exposure. We used three different markers to detect proliferating cells: 1) Ki67 expressed by the cells in all phases of cell cycle except G0 and early G1, 2) BrdU, an S-phase marker which is a synthetic nucleoside that is an analogue of thymidine, and 3) phospho-Histone 3 (pH3), an M-phase marker.

#### Ki67 labeling

Slides were washed in PBS, followed by incubation in 3% H_2_O_2_ in methanol for 10 min. Tissues were subjected to antigen retrieval with 10 mM sodium citrate (pH 6.0) at 95^0^ C for 15 min, and blocked with 5% NGS for 1 hr at room temperature. Tissues were incubated overnight with rabbit anti-Ki67 primary antibody (1:200 dilution; Cat# RM-9106, ThermoFisher Scientific, Waltham, MA, USA; RRID: AB_2341197) at 4^0^ C. The rest of the processing with Alexa 546 and Sytox green was the same as above.

#### BrdU labeling

The protocol for BrdU has been described previously [24]. Mice were injected twice with 100mg/kg body weight BrdU (5-Bromo-2’-deoxyuridine, Millipore Cat# MAB3510; RRID: AB 2314031). The first injection was 24 hrs and the second injection was 18 hrs prior to perfusion. The perfusion, tissue harvest, and sectioning was conducted in the same manner as described above. After washes with PBS and blocking with 3% hydrogen peroxide in methanol, the tissues were treated with 0.01% trypsin solution at 37^0^ C for 12 min. This was followed by additional washes and 4N HCl at 50^0^ C for 20 min before blocking for 1.5 hrs with PBS mixed with 1% triton X-100 and 10% NGS at room temperature. The sections were then incubated in 1:500 dilution primary monoclonal, anti-mouse BrdU antibody (Cat# G3G4, Developmental Studies Hybridoma Bank, Iowa, IA, USA; RRID: AB_2618097) overnight at 4^0^ C. After three, 20-min washes in PBS, the sections were incubated in secondary antibody (biotinylated goat anti-mouse IgG, Cat# BA-9200, Vector Labs, Burlingame, California, USA; RRID AB_2336171) in 1:1000 dilution and the signal was amplified with ABC solution (Vector Labs). The signal was visualized using nickel-intensified DAB (Diaminobenzidine Kit, Vector Labs) treatment for 5–10 min. After 3 distilled water washes, the slides were then counterstained with hematoxylin and passed through a gradient of alcohol (50%, 70%, 95% and 100%) followed by two rinses in xylene and cover-slipped with Permount.

#### pH3 labeling

The protocol was the same as for Ki67 except the methanol step was omitted. Blocking was done using 5% NGS for 2 hr at room temperature. Rabbit pH3 primary antibody (Cat# 06–570, Millipore Sigma, Billerica, MA, USA; RRID: AB_310177) was used at 1:1000 dilution for incubation overnight at 4^0^ C. The secondary antibody (Alexa 546 goat-anti-rabbit) and the Sytox green fluorescent marker were the same as above.

### Labeling of differentiated cells

To determine whether differentiated Type II and Type III cells were affected by CYP injection, we used immunofluorescent markers for PLCβ2 to identify Type II cells and SNAP-25 to identify Type III cells.

#### PLCβ2-labeling

The slides were washed thrice in 0.1M PBS, 10 min each, followed by antigen retrieval with sodium citrate (pH 6.0) in 80^0^ C for 15 min. Blocking was done using a mix of 5% NGS, 1% BSA and 0.3% Triton-X 100 for 1.5 hr at room temperature. PLCß2 primary antibody (rabbit anti-PLCß2, Cat# sc-9018, Santa Cruz Biotechnology, Dallas, TX; RRID: 2163248) was used at 1:200 dilution for incubation overnight at 4^0^ C. The secondary antibody (1:1000; Alexa 546 goat-anti-rabbit) was incubated for 2 hrs in the dark. Sytox green was used as a nuclear marker.

#### SNAP-25 labeling

The protocol was the same as above without any antigen retrieval technique (protocol courtesy of Dr. L. Barlow, University of Colorado School of Medicine, Aurora, CO). The primary antibody (rabbit anti-SNAP-25, Sigma Aldrich cat # S9684; RRID: AB_261576) was used at 1:5000 dilution overnight at 4^0^ C. The concentration, time of treatment of secondary antibody and Sytox staining procedure were the same as above. Because of lack of SNAP-25 immunoreactivity on day 16 and the recent report stating a longer lifespan of Type III cells [28], we included an additional time point of day 21.

### Image analysis and cell counting

Images were captured using a Nikon Eclipse E600 Scope fitted with a color camera (Spot RT KE Diagnostic Instruments Inc.) and Spot acquisition software (Spot Advanced, Version 4.6). Where necessary for better visualization, images were imported into Photoshop 6 where image intensity and contrast were increased, and then sharpened as needed. Counting was done by observers blind to the test conditions using the criteria of Nguyen et al. [59]. Taste bud profiles in the circumvallate were selected for quantification if the taste bud had a taste pore or, if it extended across multiple sections, it was the middle or largest profile of the taste bud across serial sections, and its outer boundary (determined from Sytox green label) extended from the basement layer to the outer layer of the epithelium. Two trenches were counted for each mouse. Fungiform taste buds were selected for quantification if the taste bud had a taste pore or, if it extended across multiple sections, it was the middle or largest profile of the taste bud across serial sections, and it extended from the basement layer within the stalk of the papilla to the outer layer of the epithelium. For each cell marker, a minimum of 5 taste buds (range 5–15) were counted and averaged for each mouse, and a minimum of 25 of each type of taste bud were evaluated for each test condition.

**Fig 1:**
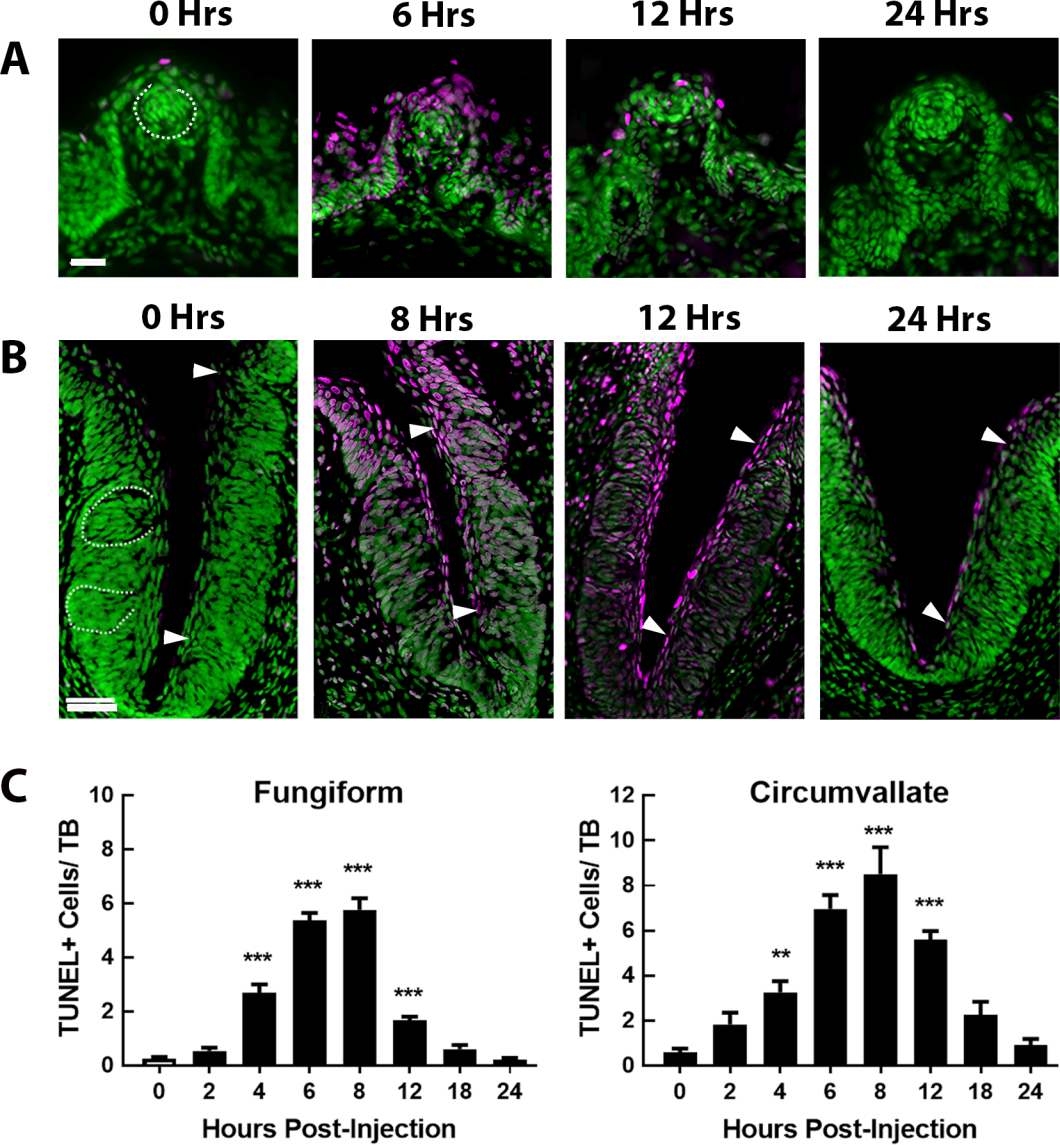
TUNEL signal in fungiform and circumvallate papillae up to 24 hrs after CYP injection. TUNEL signal (magenta) in (**A**) fungiform papillae and (**B**) circumvallate trench at 0 (control), 6, 12, and 24 hours after injection. Sections are counter-reacted with Sytox green, a nuclear marker. Examples of intact taste buds are outlined with dashed white lines in the 0 Hr images. In the circumvallate crypt, all counting occurred between the two arrows. Scale bars = 20μm. (**C**) The graphs show Mean±SEM of TUNEL+ cells/taste bud in fungiform (left graph, n = 10 mice/group) and circumvallate (right graph, n = 3 mice/group) papillae at each time point (0, 2, 4, 6, 8, 12, 18 and 24 Hrs). In both papillae TUNEL signal within taste buds was minimal at 0 hours, began to significantly increase by 4 hours post injection, peaked 6–8 hours post-injection and was near normal again at 18 and 24 hours post-injection. Time points at which TUNEL+ cells are significantly greater than control levels are identified with asterisks: * P<0.05, *** P<0.001.

For cell proliferation markers, only keratinocytes within the basement membrane underlying taste buds were counted. Examples of circumvallate counting zones can be seen between the arrows in the four images of circumvallate in Fig 1. Sections were taken from the central portion of the papilla and both sides of the trench were counted. For BrdU counting, only cells outside the border of the taste bud were counted to minimize the likelihood that these cells had entered the post-mitotic phase between the injection of the BrdU antibody and tissue collection.

The labeling index for each proliferation marker was calculated by dividing the number of immuno-positive cells for each marker (e.g., Ki67+ cells) by the total number of basal epithelial cells or taste sensory cells labeled as Sytox+, then multiplied by 100. The mean number of PLCβ2 and SNAP25 positive cells per taste bud were determined for each animal and used in all statistical analyses. The data for all experiments were collected from 3 to 6 mice per time point.

### Statistical analyses

All the statistical analyses were done with SPSS software (IBM SPSS Statistics, Version 19, IBM Corporation, Chicago, IL) and Graphpad Prism 7 software (Graphpad software, Inc., La Jolla, CA). Between-subject ANOVAs were used to detect significant changes in cell markers caused by CYP over time post-injection (e.g., days: 6 levels) and post hoc testing with simple effects tests and Bonferroni-corrected t-tests was done when applicable.

## Results

### CYP induces cell death in taste epithelium

In urinary bladder, CYP induces cell death by necrosis and apoptosis that peaks at 6 and 18 hrs post-injection, respectively [56]. To see if CYP had a similar effect on taste epithelium we used TUNEL assays to assess cell death within fungiform and circumvallate papillae of mice collected at 0 (saline control), 2, 4, 6, 8, 12, 18 and 24 hrs after CYP injection (Fig 1). Each analysis found significant changes in TUNEL+ cells per taste bud over hours post injection for both fungiform [F (7, 72) = 98.36, P<0.001] and circumvallate [F (7, 21) = 30.29, P<0.001] papillae (Fig 1C). Post hoc testing showed that TUNEL+ cells were observed infrequently in saline control mice. After CYP-injection, TUNEL+ cells in fungiform sections, including taste buds (Fig 1A and 1C), began to increase significantly by 4 hrs and peaked at 8 hrs (P<0.001). At 12 hrs TUNEL signal had decreased but was still significantly above baseline (0 hr). By 18 hrs, the number of TUNEL+ cells were again near 0 hr levels. Similarly, the number of TUNEL+ cells in the circumvallate taste buds (Fig 1B and 1C) were significantly above control levels at 4 hrs post CYP-injection (P<0.01), peaked at 8 hrs post-injection (P<0.001), and returned to baseline by 24 hrs. During the peak phase of TUNEL-reactive activity, high levels of TUNEL+ cells were visible not only in taste buds, but were also prominent throughout the non-taste bud epithelium of the surface of the tongue and in the circumvallate trench, including the basal layer of the circumvallate (see S1 File).

**Fig 2:**
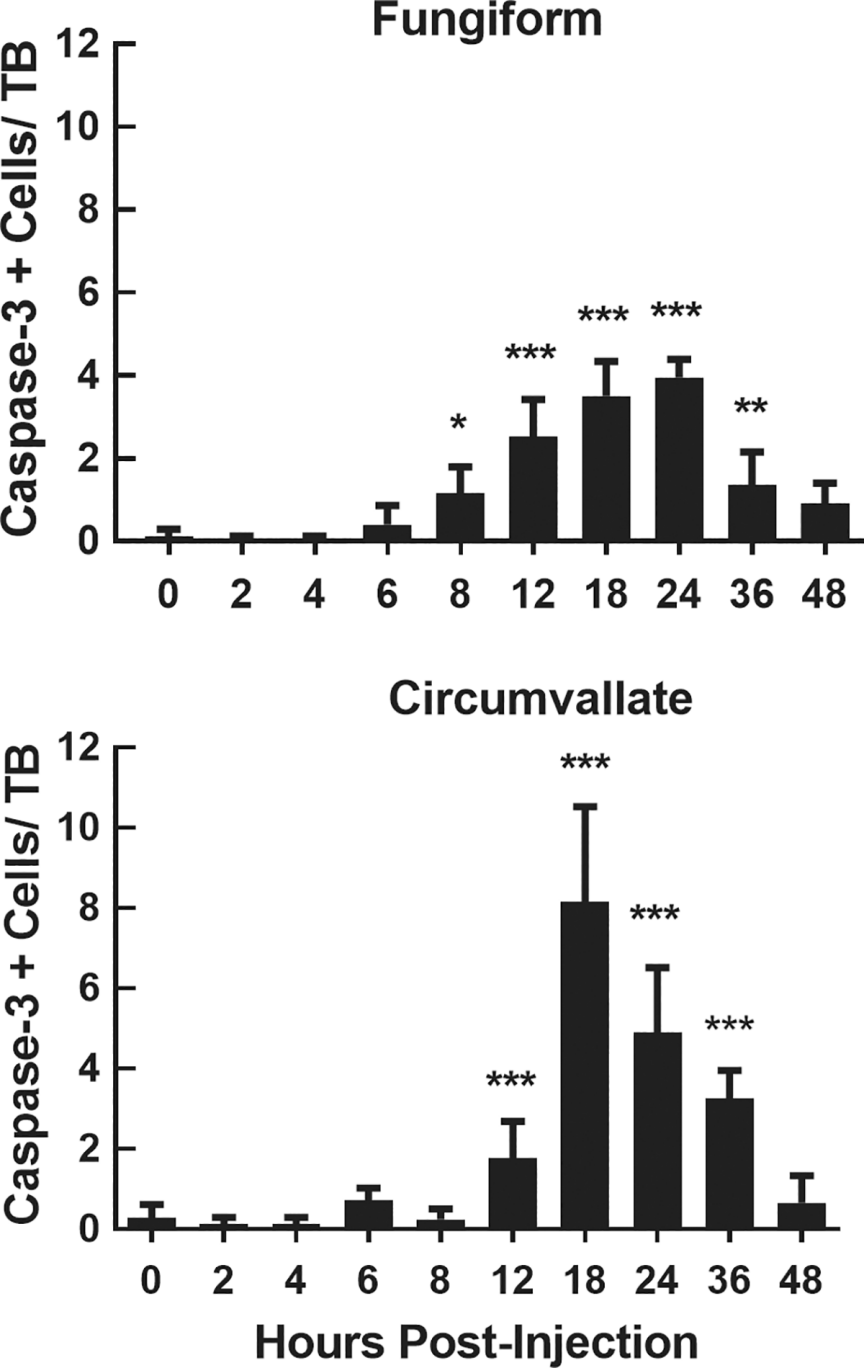
Quantification of caspase-3+ cells in fungiform and circumvallate taste buds. Mean±SEM caspase-3+ cells in taste buds in fungiform (top) and circumvallate (bottom) papillae at 0–48 hours post CYP injection (n = 4–5 mice/group). The number of caspase-3+ cells was significantly greater than control (0 hours) between 8–36 hours for fungiform taste buds and 12–36 hours for circumvallate taste buds after CYP injection. Time points at which TUNEL+ cells are significantly greater than control levels are identified with asterisks: * P<0.05, *** P<0.001.

Since TUNEL labels cells that are in the last phase of apoptosis as well as necrotic cells, we used a cleaved caspase-3 assay to identify cells undergoing apoptosis. Analysis of cell counts at 0 (control), 2, 4, 6, 8, 12, 18, 24, 36 and 48 hrs post injection showed systematic changes in caspase-3+ expression in fungiform [F (9, 38) = 28.82, P<0.001] and circumvallate [F (9, 36) = 33,58, P<0.001] taste buds over time. Saline control tissues (0 hr) revealed very few caspase-3+ cells in either fungiform or circumvallate taste buds (Fig 2). Cleaved caspase-3+ cells in fungiform taste buds significantly increased at 8 hrs (P<0.01) and reached their peak expression at 18–24 hrs post-injection (P<0.001). This was followed by a gradual decrease in labeled cells that remained significantly higher than saline controls (0 hr) until after 36 hrs post-injection. In circumvallate taste buds, the first significant increase in the number of cleaved caspase-3+ cells compared with controls (0 hr) was detected at 12 hrs post-injection (P<0.001), peaked at 18 hrs (P<0.001), and returned to near-normal levels at 48 hrs (Fig 2). These data indicate that CYP has a significant toxic effect on lingual tissues by inducing an initial necrotic phase and a later, longer lasting phase involving the cleaved caspase-3 apoptosis pathway.

### CYP targets proliferating cells of the taste system

Drugs with alkylating properties like CYP are known to target proliferating cells. Because TSCs have a relatively short lifespan and are constantly being replaced, we hypothesized that the progenitor cells responsible for the taste cell renewal cycle may be susceptible to the effects of CYP. If so, then cell cycle activity around the base of the taste bud and in the adjacent epithelial cell layer may be reduced after CYP administration.

#### Ki67 IHC

**Fig 3:**
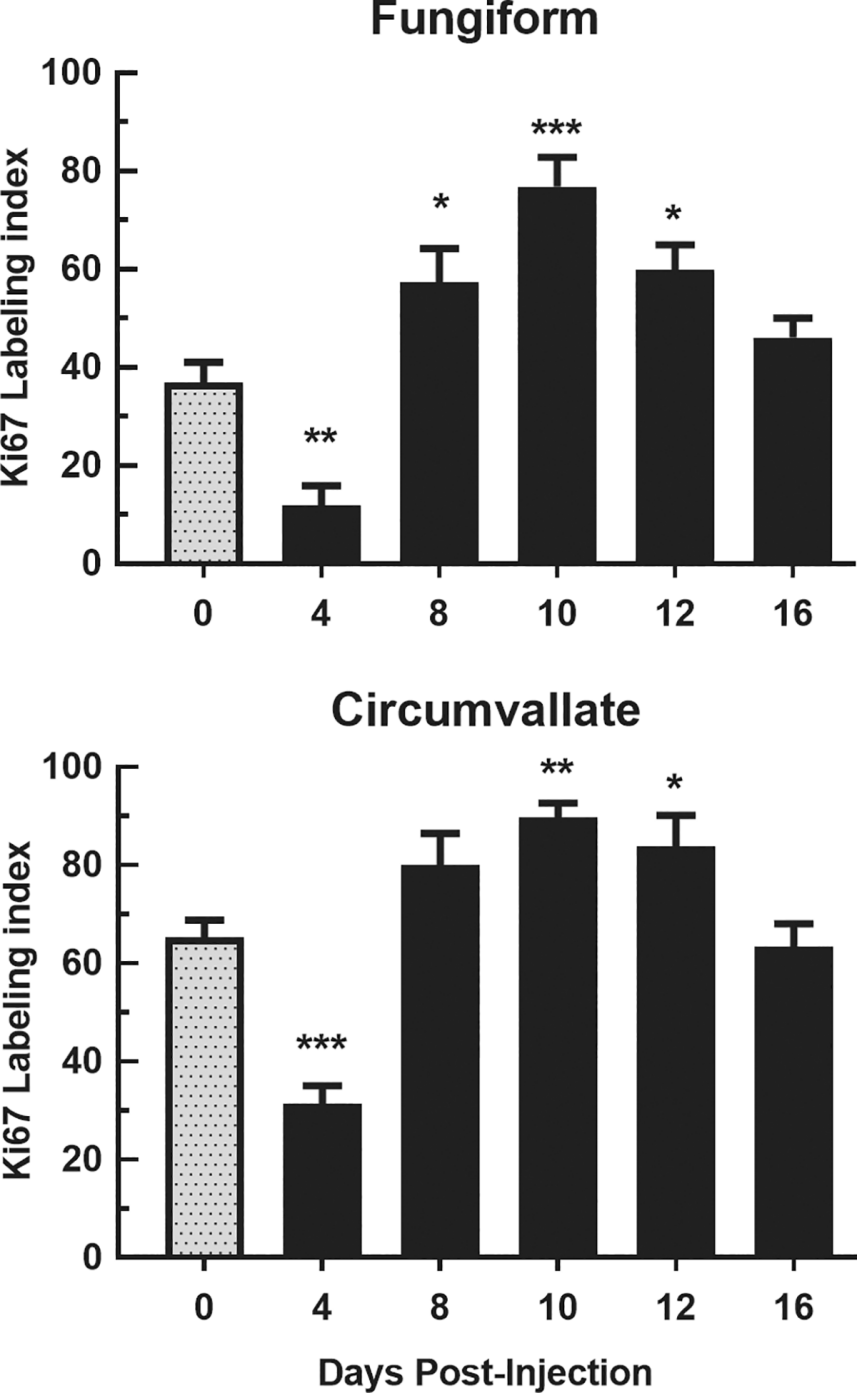
Labeling index of Ki-67+ cells/taste bud in fungiform and circumvallate papillae. Mean±SEM Ki67+ cells/total cells (labeling index) for fungiform (top) and circumvallate (bottom) papillae at 0, 4, 8, 10, 12 and 16 days post CYP injection (n = 5 mice/group). Ki67 signal was significantly lower than controls (day 0) at 4 days after CYP injection. The indexes of Ki67+ label were significantly greater than controls between days 8–12 in fungiform papillae, and 10 and 12 days in circumvallate papillae. * P<0.05, ** P<0.01, *** P<0.001.

To gain an overview of the effects of CYP on cell proliferation, we immunoreacted tissue sections with Ki67 antibody, a general marker of cell cycle activity (S1 Fig). In fungiform papillae of saline control mice, we found that about 40% of the cells in the basal layer of the taste buds were Ki67+. There were differences in the number of Ki67+ cells between the saline control and CYP-injected mice across days [F (5, 24) = 18.94, P<0.001]. Compared with saline control mice, the number of Ki67+ cells in CYP-injected mice were significantly lower on day 4 (P<0.01) but exceeded saline controls on days 8 (P<0.05), 10 (P<0.001) and 12 (P<0.05) post-injection, and returned to control levels on day 16 (Fig 3). These data suggest that cell proliferation was arrested on day 4 and then resumed with a rebound effect in the CYP-injected mice. Since the TUNEL assay showed significant cell death, it is possible that these drug effects were simply due to reduced cellular populations. Therefore, we also generated a Ki67 labeling index, calculated as the number of Ki67+ cells in the basal layer of fungiform taste bud and its immediate surrounding epithelia divided by the total number of cells in the same area identified by Sytox labeling, then multiplied by 100. ANOVA evaluation indicated that this index also varied significantly over days post-injection [F (5, 24) = 18.94, P<0.001]. The Ki67 labeling index was significantly lower 4 days post-injection (P<0.005) followed by an overshoot of control levels on days 8 (P<0.05), 10 (P<0.001) and 12 (P<0.01; Fig 3).

In circumvallate papillae (S1 Fig), we found that approximately 65% of the basal cells in controls were Ki67+. ANOVA indicated significant changes in Ki67 expression over days [F (5, 24) = 8.27, P<0.001] that mimicked the pattern seen with fungiform papillae. There were significantly lower levels of expression 4 days post injection (P<0.002) and over expression 8–12 days post injection (P<0.05) in the basal layer of circumvallate papillae. This pattern was verified by the Ki67 labeling index which was also lower in CYP-injected mice on day 4 (P<0.001) compared with saline-injected mice (Fig 3, bottom). The index then overshot baseline on days 10 (P<0.001) and 12 (P<0.05) before coming back to baseline on day 16. A similar approach was used to assess non-taste epithelium. After injection there was a significant change in the cell counts and in the Ki67 labeling index across days [F = (5, 24) = 19.31, P<0.001]. Compared with controls, Ki67+ cells in the non-taste epithelium of CYP-injected mice were decreased significantly on day 4 (P<0.001), and returned to baseline levels on later post-injection days.

#### BrdU

**Fig 4:**
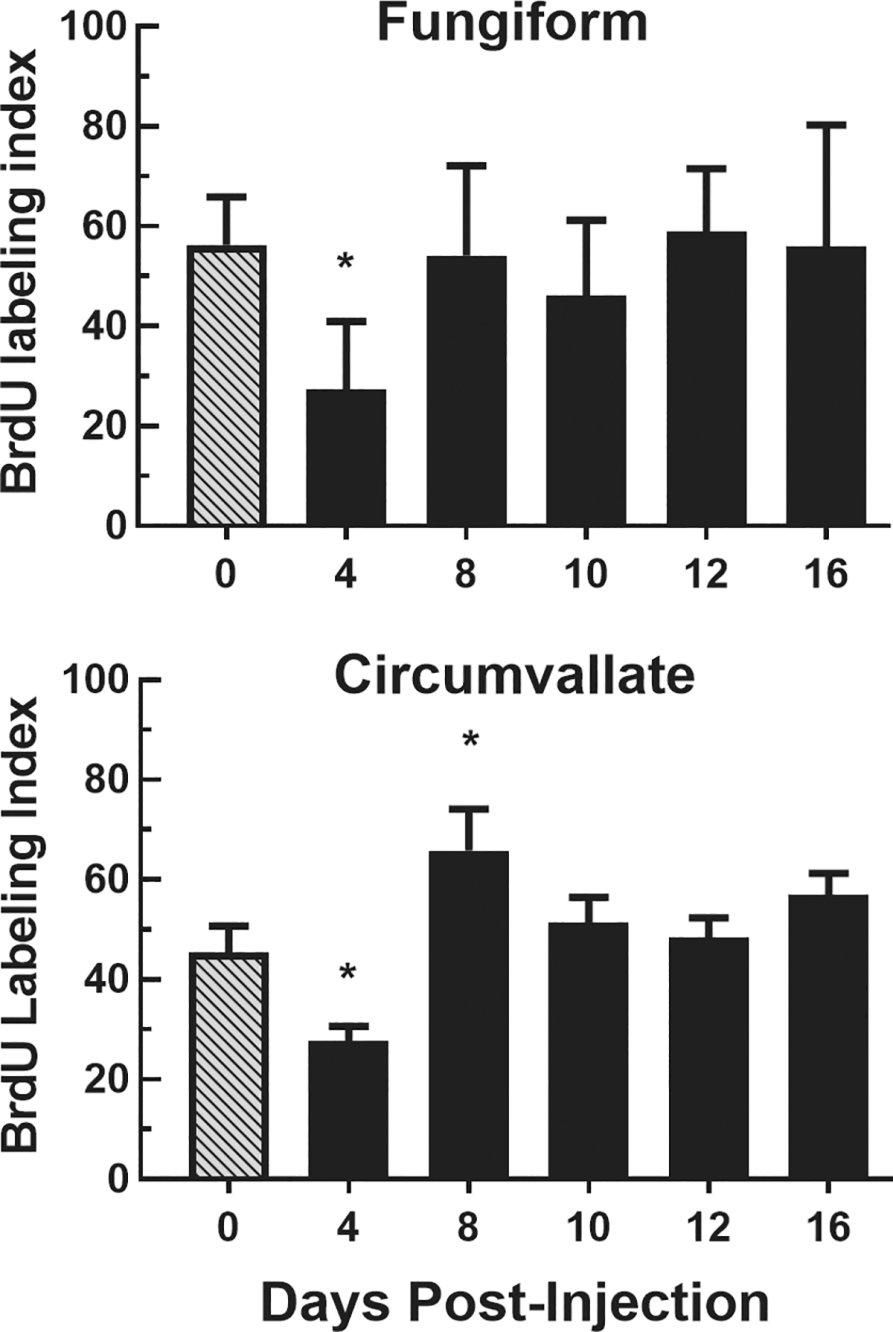
BrdU index (BrdU+ cells/total cells) in fungiform and circumvallate papillae. Mean±SEM BrdU index values for fungiform (top) and circumvallate (bottom) papillae at 0, 4, 8, 10, 12 and 16 days post CYP injection (n = 6 mice/group). The BrdU index values for both papillae were significantly lower than control (0 days) at 4 days after injection. BrdU index values for circumvallate papillae were significantly greater than controls at 8 days after CYP injection. * P<0.05.

To verify the results of the Ki67 cell proliferation experiments, BrdU was used to identify cells in the S-phase of the cell cycle. These counts were converted to a BrdU index for analysis. Significantly lower BrdU indexes were found in the basal layer of fungiform papillae of CYP-injected mice compared with saline-injected mice over post-injection days [F (5, 30) = 3.64, P<0.025; Fig 4]. Post-hoc t-tests showed the BrdU index values were significantly lower 4 days (P<0.05) after CYP injection. Similar significant decreases in the BrdU index scores for circumvallate papillae were also seen 4 days post-injection but significantly greater (P<0.05) than controls 8 days after injection.

#### pH3

**Fig 5:**
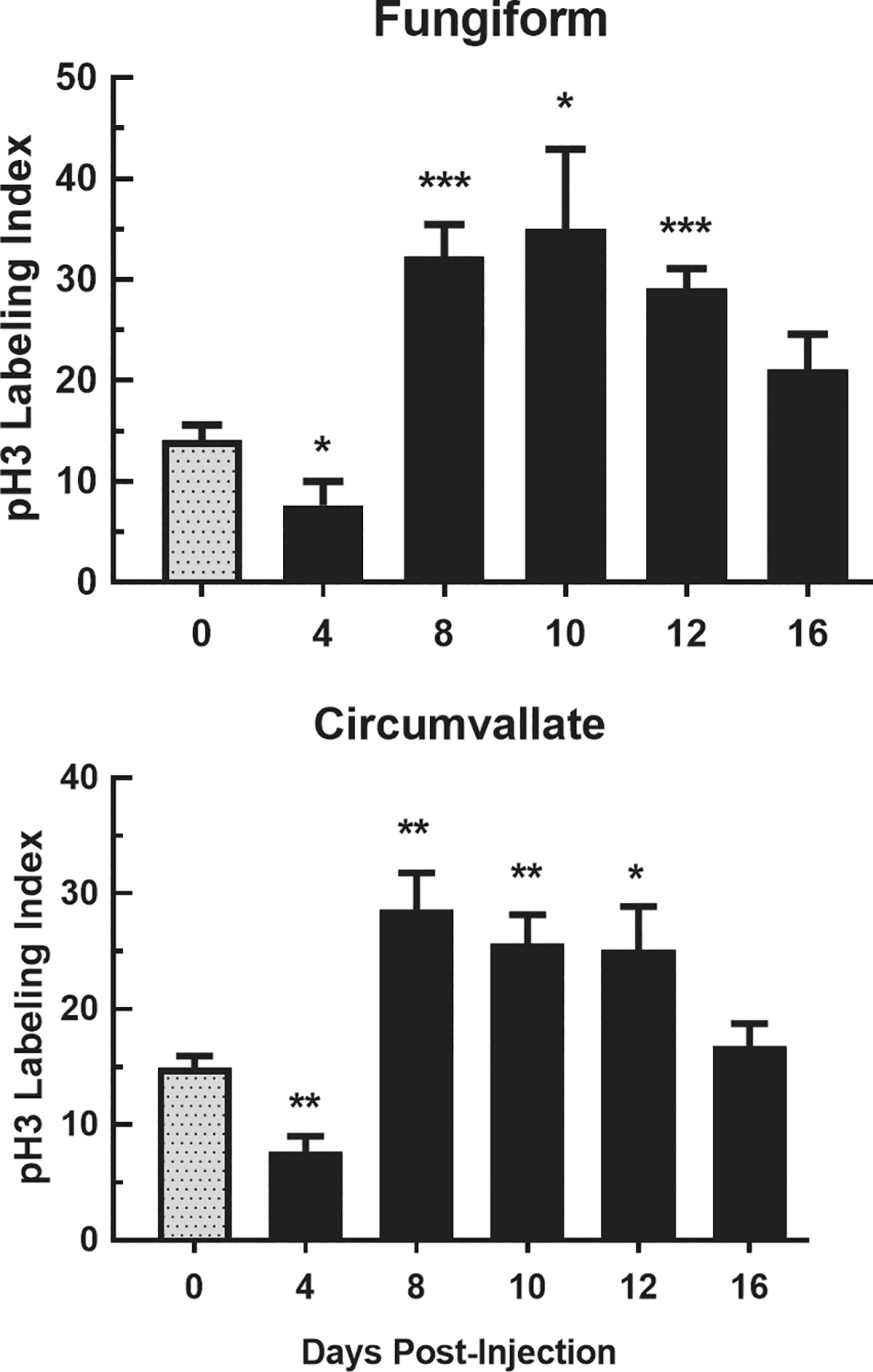
Index of pH3+ cells in fungiform and circumvallate papillae. Mean±SEM of pH3 indices for fungiform (top) and circumvallate (bottom) papillae at 0, 4, 8, 10, 12 and 16 days post CYP injection (n = 5 mice/group). The pH3 indices were significantly lower than controls (0 days) at 4 days after injection in both papillae. pH3 indices were significantly greater than controls 8–12 days after CYP injection in fungiform and circumvallate papillae. * P<0.05, ** P<0.01, *** P<0.001.

We used pH3, an indicator of M phase of the cell cycle, as a third marker to verify mitotic activity in lingual epithelium. All ANOVAs of cell counts and labeling indices indicated significant changes over days in pH3 expression in fungiform and circumvallate papillae related to CYP administration [F (5, 24) ≥ 7.33, P<0.001]. t-Tests indicated that there were significant decreases in the pH3 indices on day 4 post-CYP injection compared with saline controls. This was followed by significantly larger pH3 index values in the basal layers of both tissues on days 8, 10, and 12 (P<0.05 or less) post-CYP injection compared with saline controls (Fig 5).

### CYP affects mature TSCs of fungiform and circumvallate papillae differently

Even though the data above clearly show that mitotically active cells in lingual epithelium are susceptible to the effects of CYP, it is also important to determine if the mature, differentiated taste cells of fungiform and circumvallate taste buds are affected by CYP. If the effect of CYP on TSCs is primarily direct (i.e. cytotoxic), then there will be a decrease in the number of matured taste cells on day 4 after injection. However, if the effect is primarily indirect (i.e. proliferation of replacement cells is reduced to a level inadequate to replace differentiated cells after their natural death), then one would expect to see a decrease in the number of mature taste cells at a later point post-injection. To test these possibilities, two types of taste cells present in the taste buds were examined. Type II cells, the TSCs for sweet, bitter and umami signal transduction pathways, were identified using a marker for PLCβ2. Type III cells were labeled with a marker for SNAP-25.

**Fig 6:**
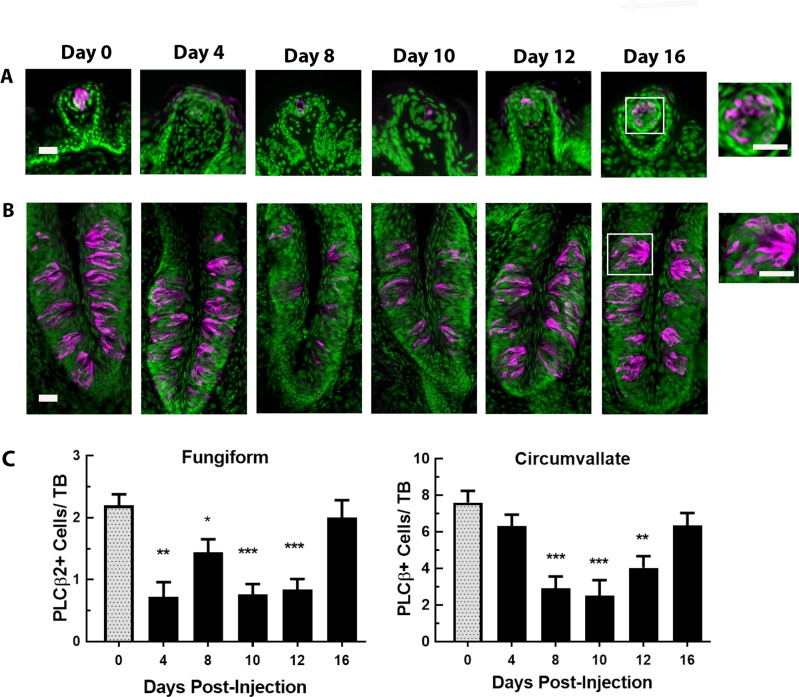
PLCβ2+ cells in fungiform and circumvallate taste buds over days after CYP injection. PLCβ2+ cells (magenta) in (**A**) fungiform and (**B**) circumvallate papillae at 0, 4, 8, 10, 12, and 16 days after CYP injection (n = 5 mice/group). Sections were counter-reacted with Sytox green, a nuclear marker. In fungiform taste buds, PLCβ2+ cells are much reduced by 4 days and do not fully recover until 16 days after injection. PLCβ2+ cells are decreased in circumvallate taste buds at 8–12 days after injection, and recover by 16 days. Scale bars = 20μm. (**C**) Mean±SEM PLCβ2+ cells per taste bud in fungiform (left graph) and circumvallate (right graph) papillae. In fungiform taste buds, the number of PLCβ2+ cells were significantly lower than controls (0 days) at 4–12 days after injection. In circumvallate taste buds, the number of PLCβ2+ cells did not decline significantly compared to controls until 8–12 days after CYP injection. * P<0.05, ** P<0.01, *** P<0.001.

In fungiform taste buds, PLCβ2+ cells were readily detected in the taste buds of saline control mice (Fig 6A and 6C left graph). However, CYP significantly decreased this population [F (5, 24) = 9.40, P<0.001]. There were significantly fewer PLCβ2+ cells/taste bud in CYP-injected mice compared with controls on days 4–12 (all Ps<0.05 or less). The number of PLCβ2+ cells/fungiform taste bud in CYP-injected mice did not recover to control levels until day 16.

Examination of circumvallate taste buds revealed a different effect of CYP on the PLCβ2-positive cells (Fig 6, right graph). There was significant difference between the saline and CYP-injected groups across days [F (5, 29) = 9.270, P<0.001]. On day 4 after CYP-injection, there was no significant change in the number of PLCβ2+ cells per circumvallate taste bud. There was, however, significantly fewer PLCβ2+ cells per taste bud at days 8–12 post-injection (Ps<0.01 or less). These data suggest that Type II taste cells of fungiform and circumvallate taste buds are affected differently by CYP. Type II taste cells in fungiform were affected within 4 days after injection and did not show recovery until 16 days post-injection. In contrast, in circumvallate taste buds the number of Type II taste cells did not decrease until 8 days post-injection and did not recover until 16 days post-injection.

**Fig 7:**
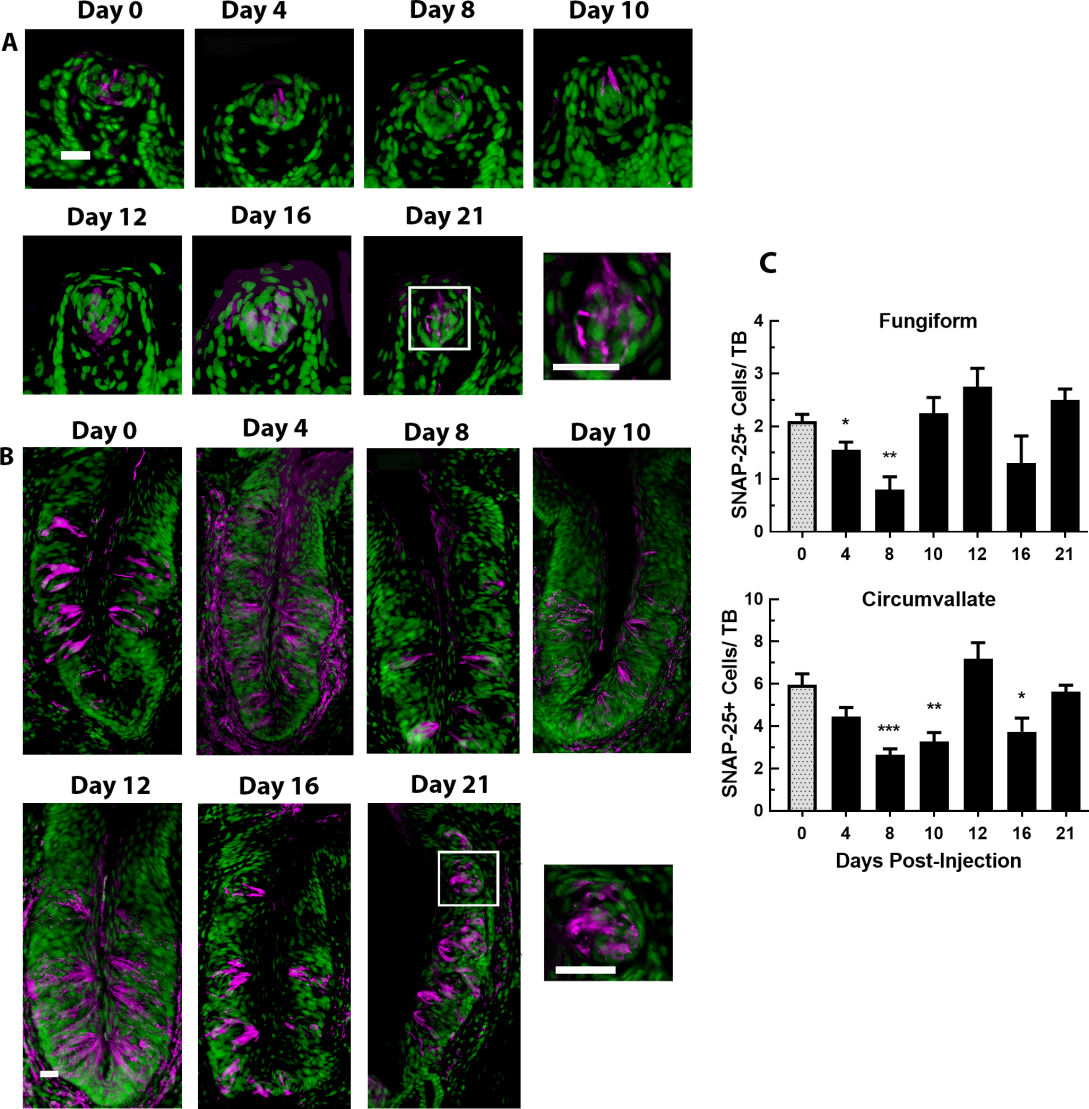
SNAP-25 signal in fungiform and circumvallate papillae over days after CYP injection. Images of SNAP-25+ cells (magenta) in (**A**) fungiform and (**B**) circumvallate taste buds at 0, 4, 8, 10, 12, 16 and 21 days after CYP injection (n = 4 mice/group). Sections were counter-reacted with Sytox green, a nuclear marker. In fungiform taste buds, there are fewer SNAP-25+ cells by 8 days after CYP injection, then they appear to recover. In circumvallate taste buds, SNAP-25+ cells are decreased at 8–10 days after injection. This cell type appears to recover by 12 days post-injection but then decreases again 16 days post injection. They return to control levels by 21 days after injection. Scale bars = 20μm. **(C)** Mean±SEM SNAP-25+ cells per taste bud in fungiform (left graph) and circumvallate (right graph). In fungiform taste buds, the number of SNAP-25+ cells were significantly lower than controls (day 0) at 4–12 days after CYP injection. In circumvallate taste buds, the number of SNAP-25+ cells did not decline significantly compared to controls until 8–12 days after CYP injection. * P<0.05, ** P<0.01, *** P<0.001.

Type III cells were also examined with the antibody marker for SNAP-25 (Fig 7). In fungiform taste buds (Fig 7A and 7C left graph), there was a significant drop in the number of SNAP-25+ cells/taste bud of CYP-injected mice compared with controls [F (6, 21) = 5.490, P<0.01], specifically on days 4 (P<0.05) and 8 (P<0.001) post-injection. In circumvallate taste buds (Fig 7B and 7C right graph), there were significantly fewer SNAP-25+ cells/taste bud on days 8 (P<0.001), 10 (P<0.01), and 16 (P<0.05) post CYP-injection compared with controls [F (6, 27) = 10.03, P<0.001]. The number of SNAP-25+ cells/taste bud again came back to control levels on day 21.

## Discussion

Patients undergoing chemotherapy often report changes in taste sensitivity or perception of taste sensations [1–9]. Even though these disturbances in taste functions are linked to poor dietary intake, poorer prognosis of treatment, and lower quality of life, they are not viewed as life-threatening symptoms and have often been overlooked in the clinical and scientific literature. Long-standing explanations of these disturbances have invoked learning and cognitive factors that result in the development of conditioned taste aversions [21, 60]. Because chemotherapy drugs such as CYP, an alkylating agent, attack open DNA strands, it is not surprising that research is revealing biological disturbances as well. In mice a single moderate dose of CYP caused a two-phase disturbance in behavioral measures of taste function, the first occurring immediately after injection and lasting 4–5 days, and the second occurring 8–15 days after drug administration [23–25]. Here we explored the cellular mechanisms underlying CYP-induced taste loss in mice by extensive evaluation of both fungiform and circumvallate taste buds.

The data reported here indicate that the cytotoxic effects of CYP impact the taste system and taste functions in at least two critical ways. One way (direct) causes a loss of lingual epithelium, including some gustatory epithelium, which results in an immediate disruption of taste function. Earlier findings suggested that these effects are enough to cause a reduction in fungiform taste buds within 2–4 days after CYP administration [24]. The second way (indirect) causes an interruption of normal taste cell replacement process that results in a later disturbance in taste function when aging taste sensory cells die without replacement. Immediately after administration, CYP causes cell death in two stages, the first of which peaks at 6–8 hours and returns to baseline within 24 hours of injection (TUNEL assay) and the second begins about 12 hours, peaks at 18–24 hours and returns to baseline between 36–48 hours (caspase-3 assay). These data align with previously published work showing that CYP caused necrosis and then apoptosis in the bladder that peaked at 6 and 18 hrs, respectively, after injection [56]. These data are also comparable to data showing that the combination of tamoxifen and irradiation of Lgr5 and Bmi 1 intestinal cells induced a rapid form of apoptosis [61]. TUNEL+ cells peaked 6 hr after irradiation, coinciding with the time course of apoptosis associated with the cleavage of caspase-7, caspase-9, and poly (ADP-ribose) polymerase, but not with the caspase-3 pathway [61]. Since TUNEL assay only detects fragmented DNA, regardless of the cause, it is unclear whether the initial period of cell death in this study is the result of direct cytotoxic effects and/or the initiation of a rapid form of apoptosis or both. However, by 18 hours post-injection, the cause of cell death appears to shift predominantly to the initiation of the canonical apoptotic pathway involving cleavage of caspase-3 in cells unable to recover from the effects of CYP. These findings suggest that cellular loss within the gustatory epithelium is initially due to direct cytotoxic effects followed by a period in which cells struggle to survive.

Activity associated with cell cycle and cell proliferation was significantly reduced at 4 days following CYP injection, confirming previous findings [24]. Alkylating agents like CYP attack proliferating cells, damage their DNA and arrest cell cycle activity [62]. Under normal conditions taste cells have relatively short lifespans, depending upon cell type, and are continuously lost and replaced. The source of replacement cells is in the basal layer just inferior to taste buds [30, 32–35] where transit amplifying cells undergo rapid mitosis and are natural targets for chemotherapy agents such as CYP that attach open DNA strands. Our findings that cell cycling is arrested in taste epithelium at 4 days post-injection are consistent with this hypothesis. Interestingly, after proliferation resumed, the proportion of cells in S and M phase increased to levels higher than controls on days 8–10 post-injection. These findings are similar to those observed after irradiation of taste epithelium [59], possibly due to production of additional transit amplifying cells, and/or a shortening of the cell cycle for transit amplifying cells [63, 64]. However, mechanism underlying this rebound effect is not known, but may involve signal pathways such as sonic hedgehog [65–68], Wnt/β-catenin [34, 69–71], Lgr5, Lgr6 [72–74], and other related molecular mechanisms [31, 35, 75].

While proliferating cell populations are prime targets of CYP, the populations of mature Type II and Type III TSCs were also adversely reduced by CYP. Although the onset of the reduction of each cell type was dependent on the location of the taste buds, Type II and Type III taste cells returned to control levels by 16 and 21 days, respectively, after CYP-injection. One might then ask how this time frame compares with the timing of taste cell differentiation after the cell emerges from the progenitor pool. Post-mitotic precursors enter taste buds within 12–24 hrs [36, 37], and differentiation of Type II taste cells is thought to be complete between 3–6 days after birth [76, 77]. Assuming the average lifespan of a Type II taste cell is 8 days [28–31, 33] and that taste buds in mice are comprised of approximately 60–100 cells [27], then under normal conditions several older cells will be lost each day and a similar number of new cells will be added to maintain the taste cell complement within a taste bud. Our data suggest a model in which, after a single dose of CYP, significant loss of differentiated cells such as Type II cells is not readily detectable until they die at the end of their natural lifespan and a shortage develops of taste sensory cells needed to maintain the functional population within a taste bud. Loss of these mature cells cumulates with each day and appears to peak around 8–10 days following CYP injection. By 16 days post-injection, new cells born during the post-injection regeneration phase (5–8 days) have migrated into a taste bud and differentiated, enabling taste buds to regain most or all of their cellular complement. Surprisingly, Type III cells were also reduced but at two different time points, 8 and 16 days post injection. It is possible that this is a heretofore unknown natural cycle of cell replacement or rates of maturation of Type III cells. It is also possible that replacement of Type III cells, with a much longer lifespan (average 22 days [28]), are regulated by somewhat different signal mechanisms than Type II cells [34] (see also review and model by Barlow [75]). Our results suggest that CYP-induced cytotoxicity may have induced a feedback cycle that generated waves or pulses of new cells that matured into Type III cells. If so, then the effects of a single exposure to CYP and other chemotherapy drugs may have longer term effects than previously thought.

Taste cells in fungiform papillae appear to be more vulnerable to the effects of CYP than circumvallate papillae. In a previous study, the number of fungiform papillae decreased significantly within 2 days of the CYP injection whereas taste buds in the circumvallate did not decline until 8 days post-injection [23–25]. Both recovered about 12 days after injection. In this study, SNAP-25 and PLCβ2-labeled cells in surviving fungiform taste buds had significantly decreased by day 4 but they did not decrease in circumvallate taste buds until day 8 post-injection. Fungiform taste buds appear more susceptible to the cytotoxicity of the drug, possibly due to one or more features related to their anatomical location, vascularization, or embryological origins. Fungiform taste buds are scattered on the anterior surface of the tongue, especially at the tip of the tongue, while the circumvallate are located at the extreme posterior end of the tongue and are enclosed in a crypt. Perhaps differences in distribution of blood or innervation patterns to these regions of the tongue may be sufficient to differentially expose fungiform and circumvallate to CYP. Another possibility is that CYP might enter the mouth with crevicular fluid. This fluid originates from plasma and is secreted from the gums into the mouth during chewing [6, 20], thus may contain the remnants of alkylating by-products of CYP. A third possibility might be related to the differential configuration of galactosyl residues in the two types of taste buds and the presence of mannose in circumvallate taste buds but not in fungiform taste buds. In addition, mucins and N-linked glycoproteins (with a hormone-like paraneuronal function) present in the circumvallate and not in fungiform, may provide protective effects against cytotoxicity in circumvallate taste buds [78]. While molecular studies have found several anterior-posterior differences in gene expression during development and putative signal mechanisms underlying taste cell renewal [31, 75], none of these has yet been shown to have a protective effect unique to one population of taste buds. Even so, there are subtle differences between the two kinds of taste papillae and one or more of these factors may contribute to the differential effects of CYP. The basis for this interesting difference in CYP-susceptibility is currently being explored.

The data reported here argue that the CYP-induced biphasic loss of taste behavior is the result of cytotoxicity that may kill normal taste sensory cells and, in particular, attacks progenitor cells that maintain cell replacement and normal taste function. However, other factors may also contribute to our findings. First, prolonged chemotherapy may lead to chemotherapy-induced neuropathy (CIPN) which can damage nerve fibers that innervate taste buds, causing taste cell death indirectly [79–81], as maintenance of mature taste cells requires nerve contact [82–84]. It is possible that neuronal fibers serving taste buds are damaged by CYP or are altered by loss of taste cells within the bud. However, to date, nerve damage has not been reported after acute treatments or to cause a bi-phasic deficit in taste function such as that seen within the time interval of the behavioral deficits reported after a single moderate dose of CYP [23, 24]. Second, chemotherapy can damage accessory structures like Von Ebner glands and salivary glands causing xerostomia or dry mouth. This leads to severe hyposalivation, which may contribute to hypogeusia [85, 86]. Previously, we found that the output of salivary glands is affected 4 days post-injection but not later, thus ruling out the possibility that the taste loss on days 8–15 post-injection is due to hyposalivation [24]. Third, there may be a drug-induced inflammatory response which could also contribute to the loss of taste function [87–89]. Inflammation activates an interferon-mediated pathway that can disrupt cell turnover in taste buds. If so, then this reaction could add to the initial direct effects of CYP on peripheral taste mechanisms. It is possible that the biological basis of each of these models contributes to the taste disturbances following administration of CYP but each model also appears incomplete, unable to account for a biphasic disturbance in taste function. Further research is ongoing to test these possibilities.

In summary, our data indicate that the detrimental effects of CYP on taste are, at least in part, due to the drug’s cytotoxic effects on gustatory epithelium, resulting in a direct and an indirect disturbance in taste function. The direct cytotoxic effects of CYP occurs immediately following administration and cause an early loss of fungiform papillae, some taste sensory cells, but the drug is particularly harsh on the population of rapidly dividing cells in the basal layer of taste epithelium. The loss of the papillae and sensory cells directly disrupts taste function immediately after administration, but the loss of the proliferative cells has a more indirect and delayed effect on taste by temporarily retarding the system’s capacity to replace normal aging and dying taste sensory cells. For instance, the population of Type II and Type III are reduced 8–14 days post CYP-injection. The timing of the loss of these taste cells is broadly congruent with our previously published behavioral data where we report elevation of detection thresholds for MSG and IMP on 2–4 days and 8–12 days post CYP-injection, and elevation in sucrose detection thresholds of CYP-injected on days 2–5 and days 8–15 post-injection [23, 25]. Thus, the combination of direct and indirect effects of a single dose of CYP reduces the population of sensory cells below a critical level needed for normal taste function. As a result, chemotherapeutic disturbances in taste functioning may pose dietary challenges at a time when the cancer patient has a significant need for well-balanced, high-energy nutritional intake.

## Supporting information

S1 File TUNEL data. TUNEL+ cells within the basement layer of the circumvallate were counted separately at 6 and 8 hours after CYP injection. Using the labeling index (TUNEL+ cells/total cells), 62.5 +/-4.1% of the cells showed evidence of TUNEL signal in CYP injected mice whereas only 8.26+/-0.56% cells were TUNEL+ in control mice (t-test, P<0.001). These data verify the vulnerability of the cells in this layer to the cytotoxic effects of a single dose of CYP.
(DOCX)

S1 Fig Ki-67+ cells in the basal layers of fungiform and circumvallate papillae. Ki67+ cells (magenta) in basement layer of (A) fungiform and (B) circumvallate papillae at 0, 4, 8, 10, 12, and 16 days after CYP injection. Tissues are counter-reacted with Sytox green, a nuclear marker. The number of Ki67+ cells are significantly reduced 4 days after injection, then rebound 8–12 days after injection of CYP before returning to control levels by day 16 post injection. Scale bars = 20 μm.
(TIF)
