# Supplementary material for: Cellular mechanisms of cyclophosphamide-induced taste loss in mice
Source: PLoS One. 2017 Sep 26;12(9):e0185473. doi: 10.1371/journal.pone.0185473 (PMC5614555; doi:10.1371/journal.pone.0185473)
Supplement: S1 File — TUNEL+ cells within the basement layer of the circumvallate were counted separately at 6 and 8 hours after CYP injection. Using the labeling index (TUNEL+ cells/total cells), 62.5 +/-4.1% of the cells showed evidence of TUNEL signal in CYP injected mice whereas only 8.26+/-0.56% cells were TUNEL+ in control mice (t-test, P<0.001). These data verify the vulnerability of the cells in this layer to the cytotoxic effects of a single dose of CYP. (DOCX) [file pone.0185473.s001.docx]

**Supplemental Materials**

**Supplemental TUNEL Data:**

TUNEL+ cells within the basement layer of the circumvallate were counted separately at 6 and 8 hours after CYP injection. Using the labeling index (TUNEL+ cells/total cells), 62.5 +/-4.1% of the cells showed evidence of TUNEL signal in CYP injected mice whereas only 8.26+/-0.56% cells were TUNEL+ in control mice (t-test, P<0.001). These data verify the vulnerability of the cells in this layer to the cytotoxic effects of a single dose of CYP.

**Supplemental Fig 1. Images of Ki-67+ cells in the basal layers of fungiform and circumvallate papillae.**

Ki67+ cells (magenta) in basement layer of (A) fungiform and (B) circumvallate papillae at 0, 4, 8, 10, 12, and 16 days after CYP injection. Tissues are counter-reacted with Sytox green, a nuclear marker. The number of Ki67+ cells are significantly reduced 4 days after injection, then rebound 8-12 days after injection of CYP before returning to control levels by day 16 post injection. Scale bars = 20 µm.
