# Supplementary figures and images for: Cellular mechanisms of cyclophosphamide-induced taste loss in mice
Source: PLoS One. 2017 Sep 26;12(9):e0185473. doi: 10.1371/journal.pone.0185473 (PMC5614555; doi:10.1371/journal.pone.0185473)

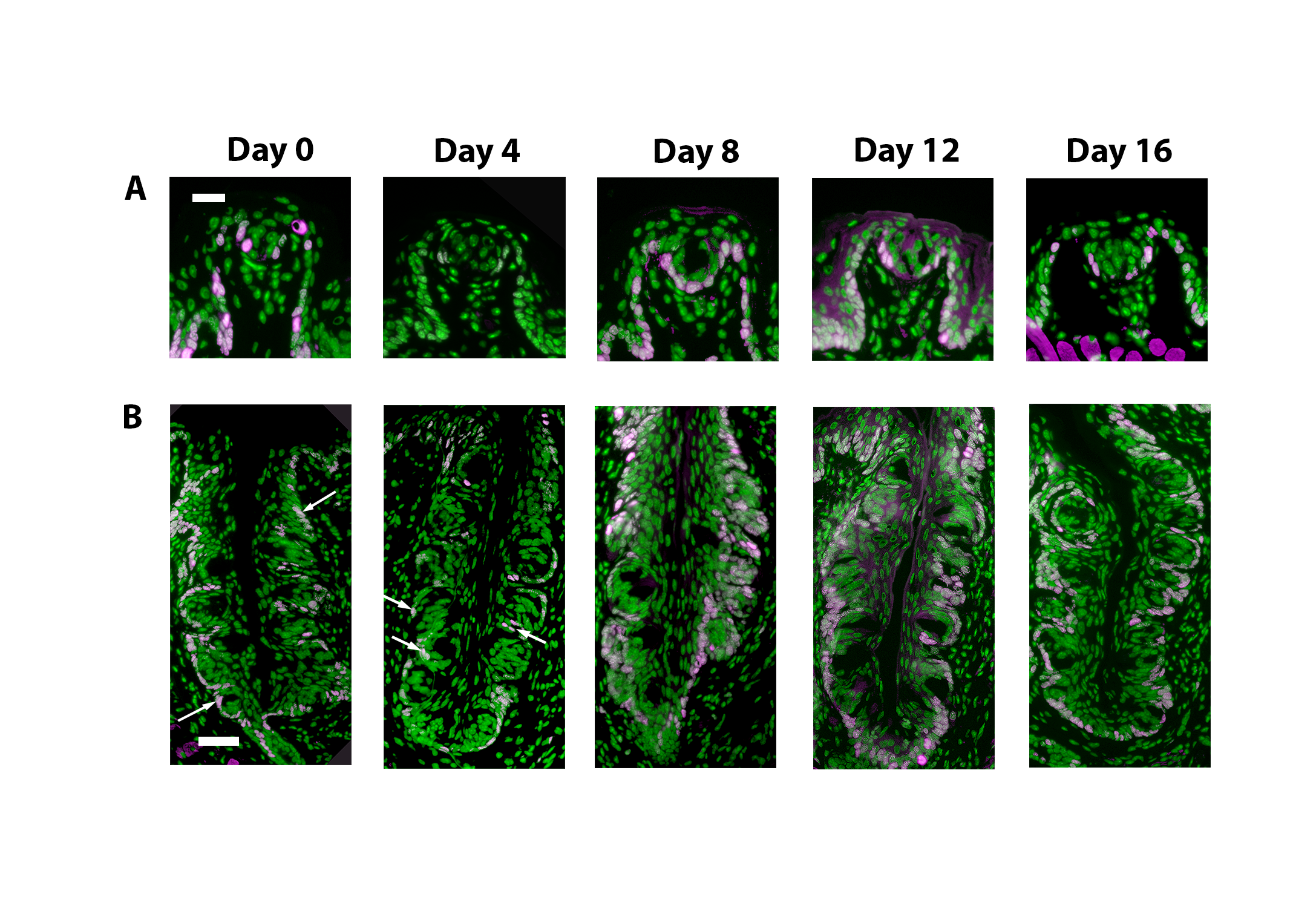

Supplement: S1 Fig — Ki67+ cells (magenta) in basement layer of (A) fungiform and (B) circumvallate papillae at 0, 4, 8, 10, 12, and 16 days after CYP injection. Tissues are counter-reacted with Sytox green, a nuclear marker. The number of Ki67+ cells are significantly reduced 4 days after injection, then rebound 8–12 days after injection of CYP before returning to control levels by day 16 post injection. Scale bars = 20 μm. (TIF) [file pone.0185473.s002.tif]
